# “I probably have access, but I can’t afford it”: expanding definitions of affordability in access to contraceptive services among people with low income in Georgia, USA

**DOI:** 10.1186/s12913-024-11133-6

**Published:** 2024-06-07

**Authors:** Anna Newton-Levinson, Kelsey Griffin, Sarah C- Blake, Andrea Swartzendruber, Michael Kramer, Jessica M- Sales

**Affiliations:** 1 Department of Behavioral, Social and Health Sciences, Rollins School of Public Health, Emory University, 1518 Clifton Road, Atlanta, GA 30322 USA; 2 Department of Gynecology and Obstetrics, Emory School of Medicine, 201 Dowman Dr, Atlanta, GA 30307 USA; 3 Department of Health Policy and Management, Rollins School of Public Health, Emory University, 1518 Clifton Road, Atlanta, GA 30322 USA; 4 Department of Epidemiology and Biostatistics, College of Public Health, The University of Georgia, 101 Buck Rd, Athens, GA 30606 USA; 5 Department of Epidemiology, Rollins School of Public Health, Emory University, 1518 Clifton Road, Atlanta, GA 30322 USA

**Keywords:** Family planning, Contraception, Access, Affordability, Health equity, Qualitative research

## Abstract

**Background:**

Disparities in rates of contraceptive use are frequently attributed to unequal access to and affordability of care. There is a need to better understand whether common definitions of affordability that solely relate to cost or to insurance status capture the reality of individuals’ lived experiences. We sought to better understand how individuals with low incomes and the capacity for pregnancy conceptualized one domain of contraceptive access–affordability --in terms of health system and individual access and how both shaped contraceptive care-seeking in the US South.

**Method:**

Between January 2019 to February 2020, we conducted twenty-five life-history interviews with low-income individuals who may become pregnant living in suburban counties in Georgia, USA. Interviews covered the ways individual and health system access factors influenced care-seeking for family planning over the life course. Interview transcripts were analyzed using a thematic analysis approach to identify experiences associated with individual and health system access.

**Results:**

Affordability was identified as a major determinant of access, one tied to unique combinations of individual factors (e.g., financial status) and health system characteristics (e.g., cost of methods) that fluctuated over time. Navigating the process to attain affordable care was unpredictable and had important implications for care-seeking. A “poor fit” between individual and health system factors could lead to inequities in access and gaps in, or non-use of contraception. Participants also reported high levels of shame and stigma associated with being uninsured or on publicly funded insurance.

**Conclusions:**

Affordability is one domain of contraceptive access that is shaped by the interplay between individual factors and health system characteristics as well as by larger structural factors such as health and economic policies that influence both. Assessments of the affordability of contraceptive care must account for the dynamic interplay among multilevel influences. Despite the expansion of contraceptive coverage through the Affordable Care Act, low-income individuals still struggle with affordability and disparities persist.

**Supplementary Information:**

The online version contains supplementary material available at 10.1186/s12913-024-11133-6.

## Introduction

Agency over one’s fertility is essential to the health of individuals and their families [1, 2]. Individuals with lower socioeconomic status, people of color, and those living in the Southeastern U.S. have lower rates of contraceptive use [3], particularly of highly effective methods [4, 5]. Lower rates of contraceptive use are frequently attributed to unequal access to family planning (FP) care [3, 6–11]. However, testing and addressing these assumptions is challenged by limitations in conceptualizing and measuring access.

“Access” has been broadly defined as the ability to seek and “to have healthcare needs fulfilled” [12]. Access encompasses the process of utilization—including an individual’s preferences about where to seek care, the process of care-seeking, the ability to reach care, as well as the use of services (realized access). Though access has long been conceptualized as involving factors pertaining to both the healthcare system and the individual, it is most frequently measured in terms of the healthcare system (i.e., provision of services) [12–14]. Access, however, is complex, consisting of interactions between both the supply (healthcare system) and demand (patient) sides [12, 14]. Levesque et al. propose a patient-centered definition of access that includes five dimensions^1^ related to the health system (e.g., cost of services) as well as corresponding abilities of individuals to access care (e.g., ability to pay), which we term *individual access factors* [12].

One key domain of access highly salient to contraceptive use and FP studies is affordability, encompassing the costs of services from providers and for consumers [12, 15]. Many studies of affordability focus solely on the health system or, in the US, on insurance status (an element of individual access) [12, 15]. Often left unexamined, “individual affordability” is the holistic measurement of both *health system characteristics* related to the direct *and* indirect costs of services, and *individual access factors* related to the ability to pay for services [12, 15].

Recent federal and state policies have renewed attention to improving the equity and affordability of FP services. The US Federal Affordable Care Act (ACA), for example, eliminated cost-sharing for contraceptive services and made it easier to get insurance through subsidization and state Medicaid expansion,^2^ thereby improving access for individuals with low-income to FP services [16]. Although studies have shown dramatic increases in insurance coverage for women since 2014, critical gaps and challenges in accessing FP services remain, particularly among low-income and uninsured women and for those living in states that did not expand Medicaid under the ACA [17–19]. In Southern states like Georgia, which did not elect ACA Medicaid expansion, there may be a greater need for publicly funded FP programs such as federally subsidized programs like Title X or state FP expansion programs [18, 19]. Georgia is one of 26 states in the US that expands FP services to low-income, uninsured women via a Medicaid Sect. 1115 waiver or a state plan amendment through a program called *Planning for Healthy Babies* (P4HB).^3^ Despite these programs, however, the number of women likely in need of publicly-funded contraception is increasing in Georgia while the number being served is decreasing at more than national rates, potentially due to shifts in funding and insurance status [20].

Thus, questions remain about the status of contraceptive access in US states without Medicaid expansion. Further, to achieve equity of access, there is growing attention to the need to better understand what access means and to assess whether common definitions of affordability relating solely to cost or insurance status are adequate. Existing studies have begun to assess the experiences of low-income people who may become pregnant and the reasons that affordability may still be a challenge but have not focused on in states without Medicaid expansion such as in the South [21, 22].

As part of a larger mixed-methods study exploring individuals’ lived experiences to more holistically define access to contraceptive services, for this qualitative study we examined the influence of both health system and individual factors on one domain of access–the affordability of services. In this paper we sought to better understand (1) individuals’ conceptualizations of contraceptive affordability in terms of health system and individual access factors; and (2) how affordability shapes low-income individuals’ contraceptive care-seeking and FP outcomes in the context of a state without Medicaid expansion.

## Methods

### Sample and recruitment

We recruited a sample of low-income, reproductive-aged individuals in suburban counties within the Atlanta metropolitan statistical area (excluding the two counties that comprise urban Atlanta proper), where public transportation and the density of publicly funded FP services are less available than in urban settings. We sought a balance of individuals who identified as Black, White, or Hispanic. We also sought a range of contraceptive care seeking experiences (e.g., private providers, community health centers, public health departments, or specialized family planning providers), as well as non-contraceptive care-seeking individuals. Individuals were classified as non-care-seeking if they had not received contraceptive services in the past three years and were not currently using a prescription contraceptive (e.g., excluding condoms, natural family planning etc.). Individuals were eligible to participate if they were: assigned female at birth^4^, aged 18–34 years, sexually active, not currently pregnant, did not wish to become pregnant in the next year, had not had a hysterectomy or sterilization, spoke English, and had an income below 250% of the federal poverty level (FPL)^5^. We focused on those aged 18–34 as they represent the majority patients using publicly funded services (80% of those in the region) [23]. Recruitment and data collection occurred from January 2019-February 2020. We employed a mix of active and passive recruitment using community-based sampling (e.g., health fairs, local businesses, colleges)—representing 8% of the final sample, clinic-based sampling (e.g., health department, Title X, & specialized FP clinics)—12% of the final sample, as well as advertising online (e.g., Craigslist)—20% and social media advertising (e.g., Facebook, Snapchat, Instagram)—60% of sample.

### Data collection

Eligible participants first completed a 15 min self-administered survey and then a life history in-depth interview (LHI) [24]. The survey assessed demographic factors (e.g., age, race and ethnicity, income) and participants’ social contexts (e.g., social support, experiences of discrimination). The lead researcher developed LHIs which assessed various factors which may influence contraceptive care-seeking over the course of participants’ lives (Appendix 1). LHI topics included health system and individual factors (e.g., health, insurance & financial status), as well as the process of seeking care (e.g., preferred care source, priorities). We asked participants who were not currently seeking contraceptive services or using contraception about their previous experiences with sexual and reproductive health (SRH) services more broadly (e.g., well woman, STI, and pregnancy care) to compare their experiences and about the contexts in which they decided to forgo care. Interviews began with participants selecting their own pseudonym and also incorporated activities to elicit additional details related to care-seeking, including free-list and timeline activities, which were referenced through the interview [24–26]. Interviews, including the survey, lasted between 60 and 90 min, and were conducted in-person by the lead researcher (ANL). Interviews were audio recorded and professionally transcribed verbatim. Participants received a USD40 gift card for participating. The study was approved by the Emory Institutional Review Board.

### Analysis

We used an iterative thematic analysis approach to identify individual and health system factors associated with affordability and their implications for care-seeking. We coded de-identified transcripts using MaxQDA 2020, (VERBI Software, 2019). The lead author (ANL) read a set of five transcripts to identify initial themes and to develop a codebook, identifying both deductive codes (e.g., ‘individual ability to pay’) derived from the interview guide based on health service access literature and inductive codes (e.g. ‘time’, ’shame’) arising from the interviews [27]. For each set of codes, two members of the research team double-coded one quarter of the transcripts to refine definitions. The research team refined codes through intercoder assessments and discussion amongst the full team. We developed themes through deep reading, annotation of coded segments, notation of patterns in the data, and finally condensing and labeling each theme [27]. The coding team discussed themes to ensure agreement and then cross-checked with interviews to ensure fit. We grouped themes according to categories associated with the research questions (individual access factors, health system factors, implications for care-seeking, etc.). We used SAS 9.2 (Cary, NC) to compute demographic survey data descriptive statistics.

## Results

A total of 800 individuals completed our screening, of which 49 were eligible. Twenty-five completed interviews (Table 1). A majority identified as Black. Less than half of the participants were employed full time. Participants had recently sought clinician-provided contraceptive care from a range of sources and six were not currently care-seeking (3 had never used contraception but had been offered it by a provider at some point in their lives).

**Table 1:** Demographic characteristics and individual access factors of individuals 18–34 who participated in a study of women’s experiences seeking family planning services in Georgia, 2019–2020

Characteristic (*n* = 25)	(%)
**Age**	
< 20	4%
20–24	32%
25–29	40%
30–34	24%
**Race/Ethnicity**	
White, non-hispanic	20%
Black, non-hispanic	56%
Hispanic, black	12%
Hispanic, other	8%
**Education**	
High school	8.3%
Some college	29.2%
Community college	25.0%
4 Year college	37.5%
**Income (FPL)**	
< 150%	32%
150–200%	40%
200–250%	28%
**Employment**	
Full-time	42.1%
Part-time	31.6%
Student	10.5%
Homemaker	5.3%
Unemployed	10.5%
**Insurance status**	
No insurance	36%
Medicaid/Peach care/P4HB	24%
Sponsored private	40%
**Currently using a (prescribed) method of contraception**	
Yes	48%
No	52%
**Most recent care-source**	
Non-care-seeking for BC	28%
Private MD	28%
Community health center	20%
SFPC	12%
Other	12%

### Thematic overview

We organized major themes into four areas: (1) individual access factors; (2) health system factors; (3) implications of a poor fit between individual and health system factors for care-seeking and FP outcomes; and (4) potential strategies for navigating a poor fit between health system and individual access factors.

### Individual access factors

Several factors influenced participants’ ability to afford contraceptive services and these factors were frequently fluctuating.

#### Instability in insurance, employment, & financial status

Most participants experienced some instability or churning in insurance status, and this often influenced their ability to access contraception. Participants reported an average of 2.5 changes in insurance in their lifetimes and most experienced at least one period of being uninsured. Insurance status changed with employment and finances and with changes in family situations, and immigration status.

*“I last had insurance in 2017. It’s been like 2 years. I changed jobs… They paid me less, but it was more out of pocket costs. Even though I knew I needed to get it I didn’t sign up for the health insurance that they offered.”* (Shirley, 30, Black).

Over their lifetimes, many participants also experienced fluctuations in their financial situations that influenced their ability to afford contraceptive care. A few participants reported having to go without care when they were laid off (made redundant) and lost their insurance. Some hurried to get their contraception before losing their insurance after a job loss. Others had to change jobs or stop working because of health issues, the need to care for family, or deaths, and these changes also contributed to changes in finances and thus their capacity to afford contraception or even their insurance.

#### Influences during youth

Access to parental insurance and/or support was a key facilitator for younger people’s (ages 15–24) FP access. Parental financial support to pay for contraception or service fees or parental help in navigating insurance processes was also seen as crucial to accessing services.

“*When I was on my mom’s insurance, my mom definitely had an influence. You know, talking to Blue Cross Blue Shield, talking to her on the phone, this, that, what accepts.”* (Nia, 26, Black).

Parental support was not always available, and many reported they did not have it when they needed it. This included participants whose parents did not have insurance or went through periods of being uninsured. One participant, Blossom, 33, Black, described how the lack of parental coverage (pre-ACA) meant that she no longer had access to contraception at age 18, resulting in an unwanted pregnancy:

*“…after I got older, I wasn’t able to afford [birth control pills], ‘cause I think back then, with the insurance– it was different. Like you[can] stay on it till you’re 26 now, but back then, it wasn’t like that… So, at 18, I didn’t have any birth control. And then, I got pregnant at 18.”*

College-based health centers were often identified as an important source of low-cost care that facilitated access when participants were in school and often had few resources.

*“The [college] health center [was] really helpful. And it’s really generalized because it has to be a catchall for the students. So I think I probably went either for a checkup…. and it was like, oh, since you did that, can I also get birth control prescription since I’m here? So that was very easy… I think to be seen it was like 10 bucks and I think the pills were probably 15 or 20… There was a time period where my family didn’t have insurance, and I think that was during that time. So it was just by being a student, the cost at the health center was pretty low.*”(Amy, 28, White)

Many felt they relied on these free services to access care when young, especially when their family did not have insurance.

#### Competing costs

Participants also spoke about other competing ‘costs’ that could influence their ability to afford contraception. These included transportation costs (e.g., for fuel, bus fare, and ridesharing) or other health related expenses (e.g., diabetes care). Participants frequently discussed “time seeking care” as a cost. This included taking time off work to attend appointments as well as time required to navigate the health system. Alexandria, 32, White, described the difficulty she experienced with having to go back every month for a prescription.

*“…that’s been tough. I don’t know why I had to go back every single month, but it was just their rule… I had to pay someone to watch my kid … Take off work, lose money.”*

### Health system characteristics

Participants cited several characteristics at both the clinic and health system levels as being associated with affordability. Participants frequently described a process of trying to find affordable care that was a match for needs that fluctuated with changes in their own lives as well as in the health system characteristics they encountered. For individuals with and without insurance, interaction with the health system was often complex. Participants who reported fewer affordability concerns usually had stable insurance (private or Medicaid) and often used the same source of care but many with insurance still struggled to afford care.

#### Co-pays & healthcare visit fees could make care unaffordable

Most participants identified ‘cost’ as being more than just the price of a method. Often participants with and without insurance reported paying high co-pays or fees for healthcare visits. Thus, while their method might have been covered, the additional visit fees were still unaffordable. Participants reported fees ranging from $15- $200, but for some even $15 could be a barrier. Thus, some individuals, especially those who did not have insurance, perceived that while they might have ‘access’ in the physical sense, they could not afford care.

*“I feel like access is about the same. Not to every facility, but I feel like it’s about the same. Funds wise it is different. I probably have access, but I can’t afford it.”* (Shirley, 30, Black, Non-Care-Seeking).

#### Varying costs

Throughout their lives, depending on where they went, individuals often encountered different costs. These costs were tied to unique combinations of individual and health system factors such as insurance status, the type and location of the service provider, the type of method desired and current insurance coverage for it, etc. For example, while some found the health department to be useful in accessing low-cost services, others found that the fees were higher at health departments or community clinics than at private providers who accepted Medicaid. Kay, 24, Black, described going to the health department with Medicaid to see if she could get contraception but discovered that they charged a copay: *“I think [the co-pay was] $35.00. And that was a lot to me at the time, ‘cause I didn’t have it.”* The private doctor she had been seeing did not charge anything, so she continued to drive an hour to the private provider for care.

Despite having Medicaid coverage Charlie, 25, Black, found that she could not get her desired method at her six-week postpartum appointment at her private provider without high cost:

*“I was like…[ok]give me the Nexplanon because I had done my research on it, and I had gotten over all the horror stories. But since I had Medicaid, it wasn’t covered… They wanted me to pay like $300 for it. I was like, I don’t have that. And I don’t want the pill. And she was like, well, you can get an IUD for free. I was still stuck on a hard no for IUD… I ended up not getting [any] birth control. […After four months I went to] the Health Department, and they offered [Nexplanon] for free…so I just got it there. But I still had Medicaid*.*”*

#### Insurance & billing processes

Individuals also encountered difficulties with clinic billing which could involve long waits or confusion to determine what the insurer would cover for a certain method. Frequently participants were informed by their service provider when these challenges (e.g., refusal of coverage, fees etc.) arose and accepted this without independently verifying. Most did not appear to know what rights they had for coverage.

*“Since I had missed the window, they had to check to see if I was pregnant because the birth control from my last session would’ve worn out by the time I returned to the doctor. And then that wasn’t covered under my insurance till the following year. ‘Cause it was the end of the year before it went over…So I had to wait a [month]to get it.”* (Kiara, 22, Black).

#### Navigating public funding for FP

The enrollment process for publicly funded FP care was often overwhelming. Enrolling in Medicaid or the P4HB program required multiple steps and forms of documentation which could be difficult and time-consuming. If individuals found themselves in places where enrollment support was offered, they were often able to gain coverage; however, some participants encountered unhelpful staff or burdensome and confusing policies that prevented them from successfully navigating the process. One participant described feeling as if she were caught in a “Catch-22”^6^ between working and needing health coverage. If she worked, she made too much to qualify for Medicaid, but could not afford insurance with her salary. Another participant described how P4HB was too burdensome to be worth using:

“*I think it’s a form of Medicaid. I never used it…They say I don’t have it, or you need to update your income…We need your check stubs. We need your lease. I just went to the health department and paid $20. It was just easier for me. With Medicaid, they’ll ask you to come up there… That’s taking off of work…You can’t get an appointment so you’re just waiting. There are people in line there for food stamps, for Medicaid, for daycare assistance. It’s an all-day thing. It wasn’t worth it.”* (Yamia, 27, Black)

Participants were also often unaware of available support programs for FP services or did not understand them. Many did not know they were eligible for P4HB or about Title X funded health centers offering low-cost services near them. Others, especially some non-citizen immigrants, were not eligible to receive benefits.

#### Medicaid & pregnancy

Further, public funding for contraceptive and reproductive care seemed only to benefit individuals when they were actively pregnant and posed even further challenges when the pregnancy ended. When participants were pregnant, they were eligible to receive Medicaid coverage during pregnancy, the process of seeking and receiving pregnancy and contraceptive care often worked well and participants described having “good care” that came with access to private doctors’ offices. Many participants compared the differences between receiving Medicaid-supported pregnancy care and the FP care they received other times at community health services. With the former, individuals were able to establish trusting relationships with (often private) providers who they said gave quality care in settings that were appealing and not overcrowded, while FP care often meant less appealing experiences at community health services.

Disenrollment from pregnancy Medicaid, which at the time of the study occurred at 60 days after delivery per Georgia state policy, frequently meant further challenges in obtaining affordable contraceptive care. This postpartum transition caused difficulties for many individuals depending on where they received care, as well as with the level of assistance they had in navigating the next steps. Some participants experienced gaps in insurance coverage or lost coverage completely and were not enrolled in P4HB, despite likely eligibility. Gaps in coverage meant that individuals missed opportunities to receive postpartum contraception, and thus prevent future pregnancies. The six-week postpartum check-up was often tenuous. If an individual missed this appointment, they then lost the ability to get coverage for contraception since their Medicaid ended soon thereafter. Participants described being delayed in receiving their follow-up appointment as a result of emergencies, needing to take time to research their method, or because of issues with the cost of the method at a certain provider. An apartment fire rendered Goldie, 25, Black, unable to keep her original follow-up appointment and thus unable to pay for the removal of her c-section staples or for contraception, and so she was forced to go without either:

*“I tried to explain to them what happened… They still pushed me back ‘cause, I guess, “Now that you’re not having no baby no more, [they] need to put pregnant people first in here.” […so they delayed the appointment]. And by the time I went back, I didn’t have Medicaid…I still have staples in my stomach. I’ve been plucking ‘em out one by one, myself… They wanted to charge me [$300 to take out the staples]. So I asked them would they be able to bill me later, and they were looking at me like I was crazy…I’m not working right not. I have no income… I did ask them how much it was just to get the birth control. They said I couldn’t do anything until I get the staples taken out*.*”*

These struggles led several participants to say that the ‘government’ doesn’t care about you unless you are pregnant.

*“I think sometimes the state thinks it’s cheaper for her to have a baby, that way it’s just another mouth to help feed and all that and it’s another statistic, another tax write-off than to actually give her the birth control she needs.”* (Sadiyah, 33, Black).

### Poor fit between individual and health system

**Fig. 1:**
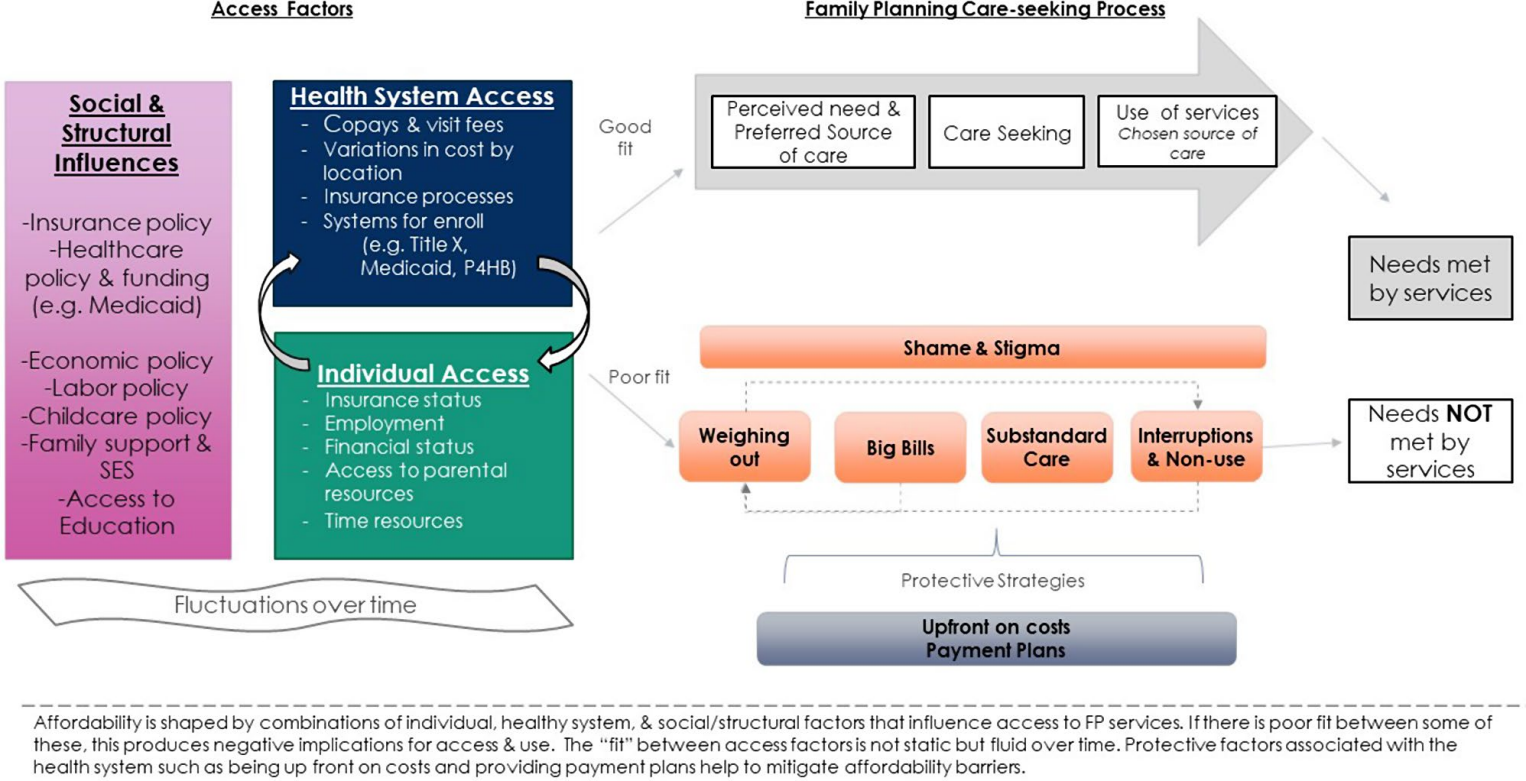
Defining individual affordability of family planning services and implications for care-seeking

Affordability, therefore, was something that changed over a lifetime and was often unpredictable, sometimes involving an intensive process of trial and error. Thus, affordability involved multiple factors associated with the health system (e.g., access to programs, accepting insurance) and individual ability to pay (e.g., financial/job/insurance status, knowledge of how to navigate insurance). “Good” affordability was determined by an alignment of health system and individual factors that met an individual’s needs in a particular moment. A mismatch or ‘poor fit’ produced inequities in access and contraceptive outcomes in several ways. The unpredictable nature of navigating affordability for FP care had implications for individuals’ care-seeking and outcomes in several ways. The relationship between health system and individual factors and the implications of poor fit between them (in orange) is shown in Fig. 1.

#### “Big, unexpected bills”

As a result of poor fit, many participants encountered large and unexpected bills associated with their contraceptive care. Participants described how a process of ‘trial and error’ involved in getting affordable care could be precarious financially. When seeking care, participants might try a new place and were often not able to get up front information about costs or insurance acceptance and would then be faced with large and unexpected bills as a result. Many participants indicated that they were still paying off these expenses.

“*I went…thinking that maybe I can establish myself with a gynecologist and I can keep going back. I was diagnosed with HPV, and I was slapped with a $1,200 medical bill… even though I have insurance because I just can’t afford [going to the gynecologist] …It’s like I need birth control, but would I be able to afford it considering me having to pay for my master’s degree out of pocket and other things*.” (Shay, 22, Black).

#### “Weighing out” immediate vs. long term costs

For some participants having no insurance or being in a difficult financial situation meant they had to “weigh out” the costs of using contraception against other more pressing expenses. Some participants without insurance described having to consider whether they ‘really needed’ care. Others described having to prioritize other immediate needs (e.g., rent, food, school) over the costs of contraception thereby risking a more costly undesired pregnancy in the long-term.

“*I was really broke and I didn’t want to pay the online co-pay just to have the repeat visit, which I thought was kind of a rip-off because … I’m visiting you from the laptop in my living room. [The copay was] $15, which doesn’t sound like much, but when you’re living paycheck to paycheck, it is significant…I just couldn’t justify the cost.”* (Ruby, 23, White).

#### Seeking substandard care

High costs and difficulty navigating insurance systems also led some individuals (unknowingly) to crisis pregnancy centers (CPCs) for perceived low-cost SRH related care. Several participants described seeking pregnancy testing at CPCs because it was free and thus much less than other places (often $150). Though individuals often initially sought care at CPCs for sexually transmitted disease (STD) testing or pregnancy services, participants frequently could not distinguish CPCs as different from other contraception providers.

“*But I’d rather go to [CPC name] and get a whole bunch of STD testing for free than to go to the gynecological group, whatever it’s called, and it’s like [the Gyn is] really nice over there, but I literally cannot afford it*.” (Shay, 22, Black)

However, at CPCs participants experienced shame, misinformation about FP care including contraception, and often incomplete care such as not being able to receive STD treatments or being refused screenings. Shay for example explained,

“*Then, every time I go to [CPC] after [I tested positive for HPV], they said, “There’s no point in doing the cervical screening if you already have HPV,” which doesn’t make sense to me.”*

#### Interruptions in care & non-use

Poor fit between individual ability to pay and health system factors ultimately led to interruptions in care and non-use of services or methods. Changes in insurance coverage of methods or in approval processes could result in being unable to afford their preferred contraceptive method and having to switch methods or stop use completely. Clinic billing processes such as verifying insurance coverage also resulted in gaps in receiving contraceptives or in “giving up.” Jasmin, 24, Black, described a series of frustrating experiences trying to get a replacement contraceptive implant and having to wait for the clinic to complete insurance verification:

“*She was taking [the implant] out of my arm. And she was like, “Well, do you want to get new birth control?” and I was like, “, yeah” … But she [said], “We’ll have to run it by your insurance to see if they’ll cover this style of birth control” …it was a lot of money… like $1,200 … And then they never called me back… to see if it was approved through my insurance… So they’re literally just not running my information… So I’m just like, “Whatever. I’ll just give up.” Because I literally don’t have time to fight with these people.”*

#### Stigma and shame

Perceptions of stigma and experiences of shame^7^ associated with one’s insurance or financial status were also prevailing themes in the interviews. Shame and stigma were produced through interactions between individuals and health systems and thus were another indicator of poor fit. Participants frequently experienced stigma attached to being uninsured or being on Medicaid. This resulted in their perceiving themselves or others as having less access to care in general and having to seek care in stigmatizing environments such as places that were overcrowded and unpleasant to be in. Blossom, 33, reflected on the atmosphere of low-income clinic spaces compared to her ideal care:

*“[It] would be nice, clean, no security. Why do you need security at low-income places vs. high-income places? Are we criminals ‘cause we don’t have any money?”*

Having money and good insurance was often perceived as the gateway to good access and to quality care. Participants also felt that individuals received lower-quality care because they were low-income or on Medicaid. Individuals expressed feelings of shame as well. One participant described going to a large community health center as reminding her that she was “*poor and made a bad decision in life so that’s why you ended up here*.” Another participant began to cry as she described how bad it felt to go to a provider as a Medicaid patient:

*“I don’t know a lot of White women that grew up the way that I did, but I mean they can’t even imagine what it feels like to think that you’re bothering your doctor because you have subsidized healthcare.”*

Ultimately, participants perceived that those who were low-income were not valued by providers or were perceived as a burden on the system.

*If you have insurance that you’re paying for … you’re going to receive a different experience… I’m pretty certain that if two people go into a facility and one has Medicaid and one has [private insurance provider], then the person with [the private insurance] is going to be treated better. It crosses racial lines. You’re more valued when you’re actually paying for the service that you receive.* (Yamia, 27, Black)

### Strategies to mitigate affordability challenges

Participants also described strategies they viewed as helpful in mitigating the unpredictable nature of affordability, depicted in Fig. 1 underneath the care-seeking pathway.

#### Upfront information about costs

Participants highlighted the importance of being told “up front” about what costs would be, particularly to avoid surprise bills. Many felt that providers were vague about costs of care and methods. A few participants described the frustration of having to wait to find out what the bill would be until after choosing a method or arriving at the pharmacy. Others related that they were not told about generic versions or fully covered options that could have led to paying less.

*“… I’ve been to clinics where they don’t up front tell you, oh, there’s a generic version. So, then you’re stuck, you know, thinking I have to pay $75 per month, where you can get it for, like, $10 or $12.”* (Laila, 26, Black).

#### Payment plans

Some participants related that while they didn’t expect costs to be free, they at least wanted them to be reasonable for what they felt they could afford. Several participants mentioned payment plans were helpful for high visit costs to mitigate both surprise bills and the need to “weigh out” whether they could afford care since they often did not have the money to pay immediately. It could be difficult, however, to find a provider willing offer them such a plan. Goldie, 25, Black, pointed out how such plans are an important way of promoting economic justice for people who were trying to survive,

“*I feel like birth control should be something that is an option, but it’s free or at very less cost…putting us on a “you got three months to pay this $25.00.” ‘Cause sometimes we may not have $25.00, you know? But if y’all wanna charge for something that y’all knocking us off anyway, so at least try to, some type of agreement with us.”*

## Discussion

This study sought to better understand how affordability of contraceptive services is shaped by both health system and individual access factors and how both shaped care-seeking for contraception. In this study, contraceptive affordability was shaped by a dynamic relationship between individuals’ ability to pay and characteristics of the health system. Both, moreover, were shaped in turn by larger structural factors. Policies and processes, such as those involved in verifying insurance or enrolling in programs, often proved to be barriers. Finding affordable care while having low-income required being connected to the right resources including enrollment in programs such as Medicaid or P4HB, having knowledge of how such systems worked, understanding how to maintain enrollment, finding a place that accepted the type of insurance or payment one had, and often trying multiple places before finding the right ‘*fit’* between the individual and the health system factors one encountered. A mismatch of these factors often produced inequities in access and contraceptive outcomes. Our study adds to a growing evidence base that affordability remains a frequent barrier to accessing contraceptive care and is associated with more than the pricing of services (health system) or insurance status (individual level) [21, 22].

Contraceptive affordability is often conceived of as static, but our findings suggest that it is rather a dynamic process that is shaped over time by an interplay among structural forces, healthcare systems, and the needs of the individual. Like other studies we found a high degree of insurance churning throughout individuals’ lives [21, 22]. Further for participants in our study, finding affordable care was an unpredictable process that involved trial and error and that differed according to where one went for care. Participants often found stable affordability difficult to achieve due to systemic inefficiencies and inconsistencies. Like others, we also found that this instability also yielded poor care experiences and outcomes including encountering large, unexpected costs, delays in care and ultimately gaps in care or forgoing care [21, 22].

Concerns with and experiences having to do with affordability also shaped care-seeking. We found, like other studies, that participants often changed whether they went for care due to costs [21, 22]. Affordability also had interplay with other domains of access, particularly the quality of care that participants received or felt they were able to receive.

The multi-level and dynamic nature of affordability has important implications for how we measure and conceptualize access. Person-centered care includes consideration of individuals’ unique needs and thus to ensure equity in access, person-centered approaches should also carefully consider affordability [29–31]. To better measure and equitably address affordability, researchers should consider indicators that reflect an individual’s lifetime experiences with health system factors and ability to pay for services (e.g., frequency of inability to afford care, or % of patients who felt they could afford quality care).

### Implications for sexual and reproductive health practice and policy

Our study has several implications for SRH practice and policy across many settings. It is crucial to note that structural barriers, such as those related to economics and health policy, influenced both individuals and health systems and thus greatly influenced overall access to contraceptive care. Insurance policies, for example, impacted both clinic processes as well as directly influenced individuals’ ability to pay. Labor protections, such as paid time-off and insurance benefits influenced participants’ ability to afford care. Addressing disparities in access and affordability thus must occur at multiple levels [1, 30]. While it may not be possible to always expect alignment or ‘fit’ between individual needs and healthy systems, there are several policies and practices that could help to achieve better fit more consistently and thus better support equitable access overall.

Some provisions of ACA were helpful to participants in obtaining affordable care. These included expanded coverage for young adults and the provision of full cost-sharing for contraception (which some experienced). As studies in other states corroborate method coverage did not always mean that individuals were consistently able to afford or use a method that they felt worked for them [21, 22]. Consistent with other studies, our findings also suggest clarifications are needed for both insurance companies and medical practices about what services, including contraceptive counseling and exams, are mandated without cost-sharing to reduce co-pays and fees [32, 33]. Improving health literacy is also essential to support individuals in gaining better knowledge about their rights and what coverage they are entitled to in relation to both private and publicly funded coverage for contraception [34]. Our data also support other findings that providing a longer supply (e.g.,1 year) of oral contraceptives could help to alleviate additional visit fees [35].

In Georgia and other US southern states with similar environments, several policies should be enacted to address individuals’ inability to pay for contraception. Subsidized FP programs, such as Medicaid, FP waivers (e.g., Sect. 1115 waiver), and Title X, are important to addressing inequities, but complex systems combined with challenges in individuals’ lives can mean that they do not get the help they need or are often unaware of available benefits. Improving knowledge of and the process by which individuals can engage with these systems is crucial to increasing access to FP services. Navigators or auto-enrollment processes could be further strengthened. While Medicaid coverage has now been extended to one year postpartum in Georgia, further expanding coverage for individuals below 138% FPL^8^, would allow individuals to get necessary care including access to contraception and to set up their next phase of coverage without experiencing gaps that endanger their health and well-being [36]. The majority of participants in our study did not know of or fully understand the P4HB program. An evaluation in 2017, found that the P4HB program served only half of people in Georgia who could be considered eligible and in need of FP [37]. Further investigation into ways to support engagement with public FP programs like P4HB could yield better access.

Across settings and particularly in under resourced contexts, further studies should explore the role that providers and others within the clinic (e.g., staff or patient navigators) could play as advocates for patients, particularly those with low incomes in relation to affordability. Strengthening the capacity of healthcare providers to navigate insurance and billing processes is also key. Insurance verification processes should not mean that patients are lost or unable to access contraception quickly. Further, individuals may then seek services provided by nonmedical organizations. Several studies, in addition to this one, have documented that CPCs provide no cost services and sometimes insurance navigation, though seemingly positive, when coupled with shame, incomplete and incorrect information can be harmful [38–40]. SRH providers may consider that providing helpful navigation and clear, consistent advertising of free services, when possible, are also critical to ensuring access to quality care.

Our study highlights that, when at all possible, providers should also consider ways to provide upfront information about anticipated costs so that patients can avoid big surprise bills. With the advent of online check-in and pre-visit readiness processes, new and creative ways to plan ahead for costs, billing, and coverage verification could be considered [41]. Coupled with insurance enrollment navigation, these strategies could offer patients a more comprehensive approach to ensuring they are able to have their visit covered. Future studies should consider the cost-effectiveness of these approaches to increase health system buy-in. Further new policies targeting big bills such as the “No Surprises Act” in the US are a promising start for emergency and out-of-network services but are not currently being implemented with consideration for most preventative reproductive care and are reliant on consumer complaints thus requiring further navigation of systems [42]. Lawmakers should consider how to enhance such policies to protect access and reproductive autonomy.

Offering cost estimates up front and payment plan options could also be a way to increase equity for individuals who are living paycheck to paycheck. Such plans are frequently offered for larger bills but could be impactful for smaller outpatient expenses as well. However, if such plans necessitate the involvement of collections agencies, they could be more harmful for patients overall.

Experiences of stigma and shame are particularly concerning and highlight the often-internalized inequities that low-income individuals face in seeking care. Sensitivity, structural competency, and bias training for providers and for staff, working in low-income settings in particular, could help ensure patient-centered and compassionate care [31, 43]. Incentivizing health systems to become accountable for providing patient-centered care that recognizes income-associated stigma is essential to address this and should include consideration whether the experiences of people with low-incomes or with certain insurance experience worse than others.

### Strengths and limitations

This study was unique in that it focused on the lived experiences of suburban individuals and included non-care-seeking individuals recruited outside of clinic spaces. The use of LHIs to document experiences over the life course also enabled a deeper understanding of the process involved in finding affordable care. This study, however, was limited to one, very large Metropolitan area and thus cannot be representative of all low-income individuals’ experiences. State-level policies may have varied influence on affordability experiences. Studies have also shown challenges in self-reporting insurance status [44] and likely some participants may have misidentified their insurance, particularly between Medicaid and P4HB. Despite this, individuals’ perceptions and understandings of insurance are important to document. Finally, this study only includes the experiences of patients, but understanding challenges that providers face is also essential to understanding access.

## Conclusion

Affordability is one domain of contraceptive access that is shaped by the interplay between individual access factors and by health system characteristics as well as by larger structural forces that influence both. Assessments of contraceptive affordability therefore must account for the interplay among these multilevel influences and must recognize the influence of affordability on access overall. Rather than being seen as static, affordability should be understood and measured as a fluid process. As we have shown, despite positive and important gains with ACA, affordability was highly influential on whether individuals with low-income could achieve equitable access to patient-centered care. Stigma associated with income or insurance status further marginalizes such patients and perpetuates inequities. Future research should address holistic conceptualizations of contraceptive affordability that better reflect individual’s lives in order to better achieve equity in meeting their needs.

### Electronic supplementary material

Below is the link to the electronic supplementary material.

Supplementary Material 1

Supplementary Material 2

## Data Availability

The datasets generated and/or analyzed during the current study are not publicly available due to containing information that could compromise research participant privacy and also due to the fact that permission was not sought at the time of participant interviews to share recordings or transcripts outside of the research team. For any enquiries regarding the datasets, kindly contact the corresponding author.

## Footnotes

1 Levesque et al. define 5 health services domains: Approachability, Acceptability, Availability, Affordability, Appropriateness (Quality) and 5 corresponding individual access domains: Ability to Perceive Need, Ability to Seek Care, Ability to Reach Care, Ability to Pay, and Ability to Engage.

2 Medicaid is the primary program providing comprehensive coverage of health care through government funding in the United States. Medicaid expansion. The ACA sought to facilitate states to expand Medicaid eligibility to individuals with incomes up to 138% of the Federal Poverty Level (FPL) but not all states have adopted this.

3 To be eligible for P4HB, individuals must be ages 18–44, U.S. citizens, residents of Georgia, have household incomes at or below 211% of FPL and not be eligible to receive other Medicaid benefits.

4 We identify participants as women and as individuals in this paper in attempt to acknowledge the range of gender identities that may be encompassed in individuals who are biologically female.

5 Federal poverty level (FPL) is a measure of income used by the US Department of Health and Human Services and is commonly used to determine eligibility for social support programs including Medicaid. The measure of those with income below 250% of the FPL is commonly used to identify those who may be in need of publicly funded family planning services.

6 A catch-22 is a difficult circumstance from which there is no escape because of conflicting or dependent conditions.

7 Stigma can be defined as attributing or labeling an individual as having undesirable characteristics. Stigma occurs in the public space and is manifest at a community or society level. Shame occurs at an individual level and has been defined as a negative emotion having to do feelings of failure in relation to personal or social standards [28].

8 Georgia currently extends Medicaid eligibility for women who are losing insurance postpartum, but the system is not automatic nor streamlined.

## Abbreviations

ACA: Affordable Care Act
FPL: Federal Poverty Level
FP: Family planning
LHI: Life history interview
P4HB: Planning for Healthy Babies, Georgia’s Medicaid family planning waiver program
SRH: Sexual and reproductive health
