# Supplementary material for: “I probably have access, but I can’t afford it”: expanding definitions of affordability in access to contraceptive services among people with low income in Georgia, USA
Source: BMC Health Serv Res. 2024 Jun 7;24:709. doi: 10.1186/s12913-024-11133-6 (PMC11157915; doi:10.1186/s12913-024-11133-6)
Supplement: Supplementary file 1 — Supplementary Material 1 [file 12913_2024_11133_MOESM1_ESM.docx]

# **Interview Guide: Never Care-Seeking**

| Interview ID: |  |
| --- | --- |
| Date: |  |
| Interviewer: |  |
| Note-Taker: |  |

### Introduction

*We are now going to start our interview. As a reminder we are interested to learn about your experiences preventing pregnancy and times when you decided not to get birth control services. We are most interested in learning about your journey and how you get the care your need as well as how you made decisions about when to get care and when not to.*

*I am going to ask you about birth control and birth control services as we talk today, but that doesn’t mean I think BC is the ‘right’ choice for you, we know everyone has different needs that means that everyone has different ways of preventing pregnancy that are right for them. Because we are trying to understand how to make health care services better for women we will ask about them so that they can meet a variety of women’s needs.*

*Before we begin I would like to ask your permission to record our discussion. This is so I can make sure that I accurately capture what you say and so I can listen to you rather than take notes.*

*Let’s make up a name for you to protect your privacy. We will use this name instead of your real name when we talk about your story. Remember you don’t have to answer any questions you don’t feel comfortable with and you can stop at any time. Also, please remember as we go through the interview, this is your story to tell, you are the expert here, this your life and your experiences, I am here to listen to you and to learn from you. Please feel free to share freely. You won’t offend me.*

*Do you have any questions before we begin? Ready to start?*

## Part I: Free-list Activity

*To get us started today we would like to talk about things that help or prevent you from* ***preventing pregnancy*** *when you want to. Thinking about your experiences, please list out everything that comes to mind for the following questions. Don’t worry about what it is just write as many things as you can think of. There are not any right or wrong answers.*

1. Things (people, places, resources) that make it easier to **prevent pregnancy** when I want to
2. Things that make it hard for me **to prevent pregnancy** when I want to

*Thank you for writing those*

- Tell me a bit about what you have written here

*Now just one more question:*

1. Of the things that you listed here, what’s most important for you overall? (*Circle or write in)*

## Part II: TimeLine

*Now we are going to talk about your experiences over your life.*

*To help us in our discussion, we are going to use a timeline to show important experiences you have had related to preventing pregnancy and sexuality.*

*Please draw a timeline of your experiences* ***preventing pregnancy*** *through your life.*

*You can start with the first time you learned about sex or had sex, and finish at this point in your life. Include any times in between that were significant for you or when something changed in how you engaged in sex or protecting yourself or felt about pregnancy.*

*You can include how you learned about sex and/or birth control, when you began sexual activity, ways you have prevented pregnancy and protected yourself and your partner when you were sexually active (like using condoms, douching, herbs etc.).*

*You can draw your timeline however you would like to, there is not a correct way, I have some examples here too if that is helpful. Please also include any other things or events that were going on during those times that you think were important or relevant—for example things that influenced what you did, or where you got services or what you were looking for when you went. This can include things like health issues, sexual activity, jobs or school, living situations, pregnancies, side effects, issues with friends/family/partner, etc.*

*Please also include any times that you thought about getting services and decided NOT go. If you decided to use other types of places or sources to get help preventing pregnancy (Like getting medications or herbs from a mail order, or talking to a friend or community member about what to do) you can put those in too.*

*Remember this is your story so include any other big events or things going on in your life that you think are important to share. We will have you draw your timeline and then we will go through together and talk through it together.*

*After initial drawing🡪 Great and before we look at it together take one more look over the list of things here—is there anything else that you would like to add to your timeline from this list?*

*Are there any other things or events that were going on during those times that you think were important or relevant—for example things that influenced what you did, or where you got services or what you were looking for when you went. This can include things like health issues, sexual activity, jobs or school, living situations, pregnancies, side effects, issues with friends/family/partner, etc.*

## Part III: Life History Narrative _ Never Care Seeking

*Thank you so much for drawing that. I am now going to ask you to tell me about what you have drawn and will ask you more questions about each part of the timeline. As we go through,* ***you can add things to the timeline and we can look back at our list of things that support*** *you and/or make it hard and talk about how they came up at different times.*

1. WARM UP: Before we talk about your experiences, can you tell me in general*:*
   - How did you first learn about birth control?
   - How do you feel about birth control?
   - How important would you say preventing pregnancy is for you (now)?
2. TIMELINE: Tell me about your timeline
   - How did your feelings about preventing pregnancy change over time?

{Timeline Notes to return to:}

**Each Time:**

- - Tell me about what was going on in your life at this time
  - How did you decide this was the right method/action for you at this time?
  - Did you consider using birth control at this time?
    - Why didn’t you get services? Or Why did you decide not get services at this time?
  - What influenced your decision not to use BC services/use BC?
    - Did anyone influence your decision?
  - What else in your life influenced whether you could access care that was right for you?
  - What about the process of trying to get health care didn’t work for you?
  - How much did access issues prevent you from using BC when you wanted to?

**If visited doctor:**

1. Where did you go?
   - What type of place was this?
     - Was this a new place for you?
     - Why did you change/stay?
   - Was this the place you wanted to go?
     - Did anyone influence you when you were deciding?
   - How did you know about this place?
   - What did you consider when you were choosing where to go?
   - Looking back at the list you made, what things that you listed were important at this time?

| Access Factors | - - - Did you feel comfortable in going to get services? (Or nervous?)     - Were you concerned about what others would think?     - Did anyone support you or influence you in getting services? |
| --- | --- |

1. **Seeking care:** *“What made it easier or harder for you to go get care this time?”*
   - What from your list did you consider when you went this most recent time?
     - Of the things mentioned, what was most important or your biggest priority?

| Personal Factors | - - - Interpersonal factors (partner, friends, children/family, etc.)     - Structural (Job hours, transportation)     - Life events     - Health |
| --- | --- |
| Access Factors | - - - Availability & Ability to reach care       - How easy was it to get to your appointment?       - Was there anything that helped or made it more difficult to get there?       - How did you get to services?       - Where were the services located?       - How long did it take you?       - Did you have to make any special arrangements?     - Affordability & Ability to pay       - Did you use insurance?       - How much of a concern was the cost of the services? |
| Social Context | - - - (social support, stigma, discrimination, social norms) |

1. **Experiences while receiving care:** *“How was it when you went to get services?”*

| Access Factors | - - - Experiences with staff?     - Experiences with provider?     - Contraceptive counseling process;       - How did you decide on your method? |
| --- | --- |
| Personal Factors | - - - Life events     - Health |
| Social Context | - - - (social support, stigma, discrimination, social norms) |

1. **Outcomes & Satisfaction** *“How was your experience when you went?”*

- How was your experience when you went?
  - Did it meet your expectations?
  - How did the providers/staff treat you?
- How well would you say the services met your needs?
- Method satisfaction?
- Would you go back again?
- Would you recommend it to your friends?
- What would your ideal care look like?

1. PRESENT STATUS:
2. **Deciding NOT to go:** “*Tell me about how you decided you that getting birth control services isn’t right for you at this time”*
   - Why did you decide not to get services?
   - Why was this NOT the right time to go?
     - How does getting birth control or preventing pregnancy fall in your priorities right now?
   - Were there other things going on in your life that influenced this decision?
   - How is this different from other times you decided not to go for family planning services? (Or to use birth control)

| Personal Context | What else that was going on in your life influenced this decision?   - - - Partners     - Transportation     - School     - Health status |
| --- | --- |
| Access Factors | - - - Do you know where to go if you wanted to? |
| Social Context | - - - (social support, stigma, discrimination, social norms) |

1. **Decision making & priorities for care:**
   - How are you currently preventing pregnancy?
     - Are you satisfied with this method/behavior?
   - Where (or to whom) else have you gone to for help preventing pregnancy?
     - Why did you prefer to go here/ to this person?
     - How did you know to go to this place/person?
     - How was your experience getting help preventing pregnancy from this place/person?
   - What gets in the way of preventing pregnancy now?
   - What are your priorities right now in your life?
2. IF YOU DID WANT SERVICES NOW: *If you did go to get birth control services what would it look like?*
3. **If you did want to get BC services now:** do you know where you would go?

| Access Factors | - - - Would you feel comfortable in going to get services? (Or nervous?)     - Are you concerned about what others would think?     - Did anyone support you or influence you in NOT getting services? |
| --- | --- |

1. **Seeking care:** *“ If you did want to get care right now, is there anything that would make it easier or harder for you to go get care this time?”*

| Personal Factors | - - - Interpersonal factors (partner, friends, children/family, etc.)     - Structural (Job hours, transportation)     - Life events     - Health |
| --- | --- |
| Access Factors | - - - Availability & Ability to reach care       - Would it be easy to get an appointment?       - Would it be easy or difficult to get to the clinic?     - Affordability & Ability to pay       - Would it be easy or difficult to pay for services? |
| Social Context | - - - (social support, stigma, discrimination, social norms) |

- - What from your list do you think influences this?
    - Of the things mentioned, what is most important?

1. **Experiences while receiving care:** *“If you did want to get care right now, how do you think your experience would be in going to get these services?*

| Access Factors | - - - Experiences with staff?     - Experiences with provider?     - Contraceptive counseling process;       - How would you decide on your method? |
| --- | --- |

1. PRIMARY CARE
   - “As an adult, have you gone to get other medical services (e.g. primary care, pap smear etc.)”
   - How frequently do you use primary care or regular health services?”
   - Where do you go for primary care services?
     - How did you decide to go?
     - Where did you decide to go?
     - What were your priorities?
   - How have your experiences been different using primary care?
     - Would you go back again to this provider?
2. IDEAL SERVICES
   - What would be your ideal experience if you were to get birth control services?
   - How would you get an appointment
   - How would you get there?
   - How would you interact with staff/doctor?

## Part IV Map

- - - 1. **Geography**

Thank you for telling me about your experiences. There are lots of reasons why individuals go to get care or don’t get care—one of them is related to how far away services are. We would like to ask some questions about the location of your home and work to better understand how far women travel to get services when they want them. We are also going to ask some questions about the location of your home and work to better understand how far women travel to get birth control services and what types of transportation they use. As a reminder we will not share this information, we will only use these addresses to get information about the distance you travel and other characteristics of where you live.

**G.2 Home**: Please put a pin on the map in about the place where you currently live/stay:

*(If you do not feel comfortable you can put a pin at a cross street, but please put it close)*

**G.3 Work**: Please enter or find on the map below the location of where you spend more of your time during the day or the place where you work. If you work at multiple locations please enter the two locations you work at most frequently. If you do not work, place it where you are most of the day.

Great thank you for providing those locations. Now we would like to ask you a bit about some of the other places on this map.

1. Were you aware of these other options [showing participant map with other providers on it]
   - Have you been to any of them?
   - Anything about your experiences in the past influence where you went?
2. If you wanted to get birth control services, how far would you consider travelling to get there?
3. If you wanted to get birth control services, would you consider going to one of these providers?
   - Time, cost, quality of services
   - Anything about your experiences in the past influence where you went?

## Norms

We are almost at the end of the interview, but I wanted to ask a couple more questions.

1. Do you think your experiences are like those of other women you know?
   - Why/why not?
2. What about all women?
   - What do you think is different about their experiences?
     - Example?, Why?

## Closing

We are nearing the end of the interview today before we close I want to ask a few final questions.

- Is there anything you wish that other women could know about your story?
- What about birth control providers? What would you like them to take away from your experience?
- What about organizations that serve your community?
- Is there anything else you’d like to share with me about your story?

Free-List Activity

1. Things (people, places, resources) that make it easier to prevent pregnancy when I want to
2. Things that make it hard for me to prevent pregnancy when I want to

## Personal Sexual & Reproductive Health Timeline

Please draw a timeline of your *experiences* ***preventing pregnancy*** *through your life, starting with the first time you learned about sex or had sex and ending now.*

| **Consider Including:**   - When you learned about sex - Sexual activity - Changes in how you protected yourself form pregnancy or STDs (like using condoms, douching, pulling out) - Pregnancies - Changes in feelings about pregnancy - Any times you decided NOT to get services (or couldn’t get them) - Other places you went to get help preventing pregnancy (medications, herbs, mail order, talking to a friend or community member) | - Other health issues - Jobs or school - Living situation - Issues with family, friends, partner/boyfriend, etc. - Other life events that you think were important |
| --- | --- |
