# Supplementary material for: “I probably have access, but I can’t afford it”: expanding definitions of affordability in access to contraceptive services among people with low income in Georgia, USA
Source: BMC Health Serv Res. 2024 Jun 7;24:709. doi: 10.1186/s12913-024-11133-6 (PMC11157915; doi:10.1186/s12913-024-11133-6)
Supplement: Supplementary file 2 — Supplementary Material 2 [file 12913_2024_11133_MOESM2_ESM.docx]

# Interview Guide: Care-Seeking

| Study ID: |  |
| --- | --- |
| Date: |  |
| Interviewer: |  |
| Note-Taker: |  |

### Introduction

*We are now going to start our interview. As a reminder we are interested to learn about your experiences getting birth control services. We are most interested in learning about the process or journey of getting care, though we will also talk about what methods you have used.*

*Before we begin I would like to ask your permission to record our discussion. This is so I can make sure that I accurately capture what you say and so I can listen to you rather than take notes.*

*Let’s make up a name for you to protect your privacy. We will use this name instead of your real name when we talk about your story. Remember you don’t have to answer any questions you don’t feel comfortable with and you can stop at any time. Also, please remember as we go through the interview, this is your story to tell, you are the expert here, this your life and your experiences, I am here to listen to you and to learn from you. Please feel free to share freely. You won’t offend me.*

*Do you have any questions before we begin? Ready to start?*

## Part I: Free-list Activity

*To get us started today we would like to talk about things that help or prevent you for getting services. Thinking about your experiences with family planning services please list out everything that comes to mind for the following questions. Don’t worry about what it is just write as many things as you can think of. There are not any right or wrong answers:*

1. Things (people, places, resources) that make it easier to get birth control services when I want to
2. Things that make it hard for me to get birth control services when I want to

*Thank you for writing those*

- Tell me a bit about what you have written here

*Now just one more question:*

1. Of the things that you listed here, what’s most important for you overall when going to get birth control services? (*Circle or write in)*

## Part II: TimeLine

*Now we are going to talk about your experiences over your life.*

*To help us in our discussion, we are going to use a timeline to show important experiences you have had related to going to get birth control services. Please draw a timeline of your experiences getting birth control or getting help preventing pregnancy through your life, starting with the first time you decided to get birth control and ending with the most recent time you got it.*

*Include any times in between that were significant for you or when something changed in how you went to get services. You can draw your timeline however you would like to, there is not a correct way, I have some examples here too if that is helpful. Please include any other things or events that were going on during those times that you think were important or relevant—for example things that influenced what you did, or where you got services or what you were looking for when you went. This can include things like health issues, sexual activity, jobs or school, living situations, pregnancies, side effects, issues with friends/family/partner, etc.*

*Please also include any times that you thought about getting services and decided NOT go. If you decided to use other types of places or sources to get help preventing pregnancy (Like getting medications or herbs from a mail order, or talking to a friend or community member about what to do) you can put those in too.*

*Remember this is your story so include any other big events or things going on in your life that you think are important to share. We will have you draw your timeline and then we will go through together and talk through it together.*

*After initial drawing🡪 Great and before we look at it together take one more look over the list of things here—is there anything else that you would like to add to your timeline from this list?*

## Part III: Life History Narrative _ Currently Care Seeking

*Thank you so much for drawing that. I am now going to ask you to tell me about what you have drawn and will ask you more questions about each part of the timeline. As we go through, you can add thing to the timeline and we can look back at our list of things that support you and/or make it hard and talk about how they came up at different times.*

1. **WARM UP**: “Before we talk about through your timeline since this interview is about you and your life experiences, we want to start with a little more about you. Can you tell us a little bit about yourself and who you are?”
2. **BIRTH CONTROL & PREGNANCY**: So to start can you tell me in general:
   - How did you first learn about birth control?
   - How do you feel about birth control?
   - How important would you say using birth control is for you?
3. **FIRST TIME:**
   - Tell me about what was going on in your life the first time you decided to go for care or use BC
   - Tell me about the first time you decided to use FP services.
4. **Deciding to go:** “Tell me about how you decided you needed to go get birth control services at this time”

| Access Factors | - - - How did you know where to go?     - How did you find out about the services? |
| --- | --- |
| Social Context | - - - Did you have friends who were using BC services?     - Were you worried about anyone judging you? |

1. **Decision making & priorities for care:** “How did you decide where to go?”
   - Where did you go?
   - How did you know about this place?
   - What did you consider when you were choosing where to go?
   - Looking back at the list you made, what things that you listed were important at this time?
   - Of things mentioned what was most important, or your biggest priority?

| Access Factors | - - - Did you feel comfortable in going to get services? (Or nervous?)     - Were you concerned about what others would think?     - Did anyone support you or influence you in getting services? |
| --- | --- |

1. **Seeking care:** *“What made it easier or harder for you to go get care this time?”*

| Access Factors | - - - Availability & Ability to reach care       - How easy was it to schedule an appointment?       - How did you get to your apt? How long did it take?     - Affordability & Ability to pay       - Did you use insurance?       - How much of a concern was the cost of the services? |
| --- | --- |

1. **Experiences while receiving care:** *“How was it when you went to get services?”*

| Access Factors | - - - Experiences with staff?     - Experiences with provider?       - Was the provider you needed available?       - If not did they provide a referral?     - Contraceptive counseling process;       - How did you decide on your method?       - Did they talk about different kinds of methods?     - Did they provide any referrals if you needed them? |
| --- | --- |

1. **Personal Context:**  *Let’s talk a little about what else was happening in your life at this time?*
   - - What was happening with **work or school**?
       - - Job hours or schedule?
     - What about **partners or boyfriends**?
       - - How did they feel about you getting services?
     - Tell me about your **friends & family** at the time
     - How was your **financial situation**?
       - - How did you get around? How did you get to the appointment?
     - How was your **health**?
     - **Anything else** going on during that time that you think is important?
2. **OTHER EXPERIENCES/EVENTS:**
3. **Tell me about other experiences you have had that you feel are important?**

- What made it easier or harder to this time?
- What was different about your experiences these times?
- What from your list did you consider when you went this time?
- What was more/less important for you compared to your most recent time?
- What influenced you in trying to go

1. **Personal Context:**  *What else was happening in your life during this time?*
   - - What was happening with **work or school**?
     - What about **partners or boyfriends**?
     - Tell me about your **friends & family** at the time
     - How was your **financial situation**?
       - - Transportation
     - How was your **health**?
     - **Anything else** going on during that time that you think is important?
2. **MOST RECENT TIME**

Ok let’s talk about this most recent time. So let’s talk a bit more in-depth about this time—we are going to talk through each step of how you decided to go there and what you did to get there.

1. **Deciding to go:** “*Tell me about how you decided you needed to go get birth control services at this time”*
   - Why was this the right time to go?

| Access Factors | - - - How did you know where to go?     - How did you find out about the services? |
| --- | --- |

1. **Decision making & priorities for care:** *“How did you decide where to go?”*
   - Where did you go?
   - Was this a new place for you?
   - Did you change where you went? Why?
   - Was this the place you wanted to go?
     - Did anyone influence you when you were deciding?
   - How did you know about this place?
   - What did you consider when you were choosing where to go?
     - What were your priorities this time?
   - Looking back at the list you made, what things that you listed were important at this time?

| Access Factors | - - - Did you feel comfortable in going to get services? (Or nervous?)     - Were you concerned about what others would think?     - Did anyone support you or influence you in getting services? |
| --- | --- |

1. **Seeking care:** *“What made it easier or harder for you to go get care this time?”*

| Access Factors | - - - Availability & Ability to reach care   Take me through how you made your appointment.   - - - - How easy was it to get to your appointment?       - Was there anything that helped or made it more difficult to get there?       - How did you get to services?       - Where were the services located?       - How long did it take you?       - Did you have to make any special arrangements?     - Affordability & Ability to pay       - Did you use insurance?       - How much of a concern was the cost of the services?       - Do you remember how much you paid? |
| --- | --- |

- - What from your list did you consider when you went this most recent time?
    - Of the things mentioned, what was most important or your biggest priority?

1. **Experiences while receiving care:** *“How was it when you went to get services?”*

| Access Factors | - - - How were the staff?     - How was your provider? How did they treat you?     - Contraceptive counseling process;       - How did you decide on your method?       - Did they talk about multiple methods? |
| --- | --- |

1. **Outcomes & Satisfaction** *“Did this experience meet your expectations?”*

- How well would you say the services met your needs?
- Method satisfaction?
- Would you go back again?
- Would you recommend it to your friends?
- What would your ideal care look like?

1. **Personal Context:**  *Let’s talk a little about what else was happening in your life at this time?*
   - - What was happening with **work or school**?
       - - Job hours or schedule?
     - What about **partners or boyfriends**?
     - Tell me about your **friends & family** at the time
     - How was your **financial situation**?
       - - How did you get around? How did you get to the appointment?
     - How was your **health**?
     - **Anything else** going on during that time that you think is important?
2. Were there any times that when you decided not to go for family planning services? (Or to use birth control)
3. How frequently do you use primary care or regular health services?
   - Do you go to the same place?
   - Is it easier or harder for you to go for primary care?

## Part IV Map

1. **Geography**

*Thank you for telling me about your experiences. Now I would like to ask you a bit more about the location of your last visit. We want to learn more about where women go for care and why they choose that location. We are also going to ask some questions about the location of your home and work to better understand how far women travel to get birth control services and what types of transportation they use. As a reminder we will not share this information, we will only use these addresses to get information about the distance you travel and other characteristics of where you live.*

- 1. **Last visit:** We are going to start with the location of your last visit. We are going to use a map to help us.

Here on this map we have all of the clinics that we know of in Georgia that provide low-cost birth control services. We have the place you went most recently here [Red PIN]--- is that right?

- 1. **Home**: Please put a pin on the map in about the place where you currently live/stay:
  2. **Work**: Please enter or find on the map below the location of where you spend more of your time during the day or the place where you work. If you work at multiple locations please enter the two locations you work at most frequently.

Great thank you for providing those locations. Now we would like to ask you a bit about some of the other places on this map.

**For each place on map:**

1. Were you aware of these other options [showing participant map with other providers on it]
2. If you wanted to get birth control services, would you consider going to one of these providers?
3. Why did you choose to go to this location instead of others in the area?
   1. Time, cost, quality of services
   2. Anything about your experiences in the past influence where you went?

## Norms

We are almost at the end of the interview, but I wanted to ask a couple more questions.

1. Do you think your experiences are like those of other women you know?
   - Why/why not?

## Closing

We are nearing the end of the interview today before we close I want to ask a few final questions.

- Is there anything you wish that other women could know about your story?
- What about providers? What would you like them to take away from your experience?
- Is there anything else you’d like to share with me about your story?

Thank you for sharing your story with me today.

# Free-List Activity

1. Things (people, places, resources) that make it easier to get birth control services when I want to
2. Things that make it hard for me to get birth control services when I want to

## Personal Birth Control Service Use Timeline

Please draw a timeline of your use of birth control services starting with the first time and ending with the last time you went.

| **Consider including:**   - Where you went to get services - Type of method you got - Other health issues - Side effects - Any times you decided **NOT to get services** (or couldn’t get them) - **Other places you went** to get help preventing pregnancy (medications, herbs, mail order, talking to a friend or community member) | - Sexual activity - Jobs or school - Living situation - Issues with family/friends/partner - Other life events that you think were important |
| --- | --- |
